# Oral doxycycline prevents skin-associated adverse effects induced by injectable collagenase in a rodent model of capsular contracture around silicone implants

**DOI:** 10.1371/journal.pone.0270112

**Published:** 2022-07-06

**Authors:** Yannick F. Diehm, Dimitra Kotsougiani-Fischer, Elena Porst, Valentin Haug, Laura C. Siegwart, Daniel Overhoff, Ulrich Kneser, Sebastian Fischer

**Affiliations:** 1 Department of Hand-, Plastic and Reconstructive Surgery, Burn Trauma Center, BG Trauma Center Ludwigshafen; University of Heidelberg, Ludwigshafen, Germany; 2 Department of Radiology and Nuclear Medicine, University Medical Center Mannheim, Medical Faculty Mannheim—Heidelberg University, Mannheim, Germany; Medical University of Graz, AUSTRIA

## Abstract

**Background:**

The collagenase of the bacterium Clostridium histolyticum (CCH) is already an established treatment for fibroproliferative diseases like M. Dupuytren and M. Peyronie Although results are comparable to surgical intervention, skin laceration is a severe and relevant side effect. Doxycycline (DOX) recently rose interest as an inhibitor of matrix-metalloproteinases alongside its capabilities of skin accumulation. It therefore might be a potential skin protective agent in the use of CCH.

**Methods:**

For simulation of a fibroproliferative disease adjacent to the skin, we utilized a rodent model of capsular fibrosis involving silicone implants and subsequent fibrotic capsule formation. For in-vitro studies, fibrotic capsules were excised and incubated with 0.9 mg/ml CCH and four different doses of DOX. For in-vivo experiments, animals received 0.0, 0.3 or 0.9 mg/ml CCH injections into the fibrotic capsules with or without prior oral DOX administration. Outcome analysis included histology, immunohistochemistry, gene expression analysis, chemical collagen and DOX concentration measurements as well as μCT imaging.

**Results:**

In-vitro, DOX showed a dose-dependent inhibition of CCH activity associated with increasing capsule thickness and collagen density and content. In-vivo, oral DOX administration did neither interfere with capsule formation nor in effectiveness of CCH dissolving fibrotic capsule tissue. However, skin thickness and especially collagen density was significantly higher compared to control groups. This led to a reduced rate of clinical skin lacerations after DOX administration.

**Conclusion:**

DOX inhibits CCH and accumulates in the skin. Thereby, DOX can effectively reduce skin laceration after CCH treatment.

## Introduction

The collagenase of the bacterium Clostridium histolyticum (CCH, Xiaflex; Auxilium Pharmaceuticals, USA) is an FDA-approved drug already implemented in daily clinical practice [1]. It is used as a treatment for the fibroproliferative diseases M. Dupuytren, M. Ledderhose and M. Peyronie [2–5]. The CCH belongs to the group of calcium and zinc dependent matrix-metalloproteinases, initially isolated in 1953 [6–11]. The unique composition of CCH of type I and type II collagenases facilitates digestion of all types of collagen, except collagen type IV, the main component of the basal lamina, vessel walls and perineurium [12]. This allows safe application in highly sensitive body areas like the palm of the hand or the male genitals. Furthermore, CCH has achieved promising results in the treatment of capsular contracture around silicone implants and urethral strictures in experimental models [13–18]. CCH offers a minimal-invasive approach for the treatment of the above-mentioned constraining diseases with outcomes comparable to surgical approaches [1, 3–5, 19–21]. For M. Dupuytren, roughly 33% of patients needing intervention are treated with CCH instead of surgery [22]. Although reported side effects are generally low in severity, affection of the skin is the most common complication after CCH-injection occurring in up to 16.2% [23]. This poses a great risk for subsequent wound infection, delayed wound healing and the occurrence of tissue defects and was the leading adverse effect in the experimental models of capsular contracture, as well.

Doxycycline, a FDA-approved tetracycline antibiotic, has aroused increasing interest as an inhibitor of matrix-metalloproteinases (MMPs) and bacterial collagenases [24–27]. The CCH belongs to the group of calcium-dependent metalloproteases with other cations (e.g. zinc) mediating the enzyme´s stability, conformation and activity [24]. Besides their antibacterial properties, tetracyclines can act as chelators for multivalent metal-cations, such as calcium and zinc, and subsequently inhibit excessive collagenase activity [24, 28]. This property and the ability to accumulate in skin tissue make doxycycline a potential skin-protective agent in the setting of CCH-injections.

The purpose of this study was to investigate the inhibitory and skin-protective capabilities of doxycycline for CCH injections in an animal model. Due to the lack of an animal model for M. Dupuytren or M. Peyronie, we utilized an animal model for capsular contracture, which can adequately mimic the clinical situation of a fibroproliferative disease adjacent to the skin.

**Fig 1:**
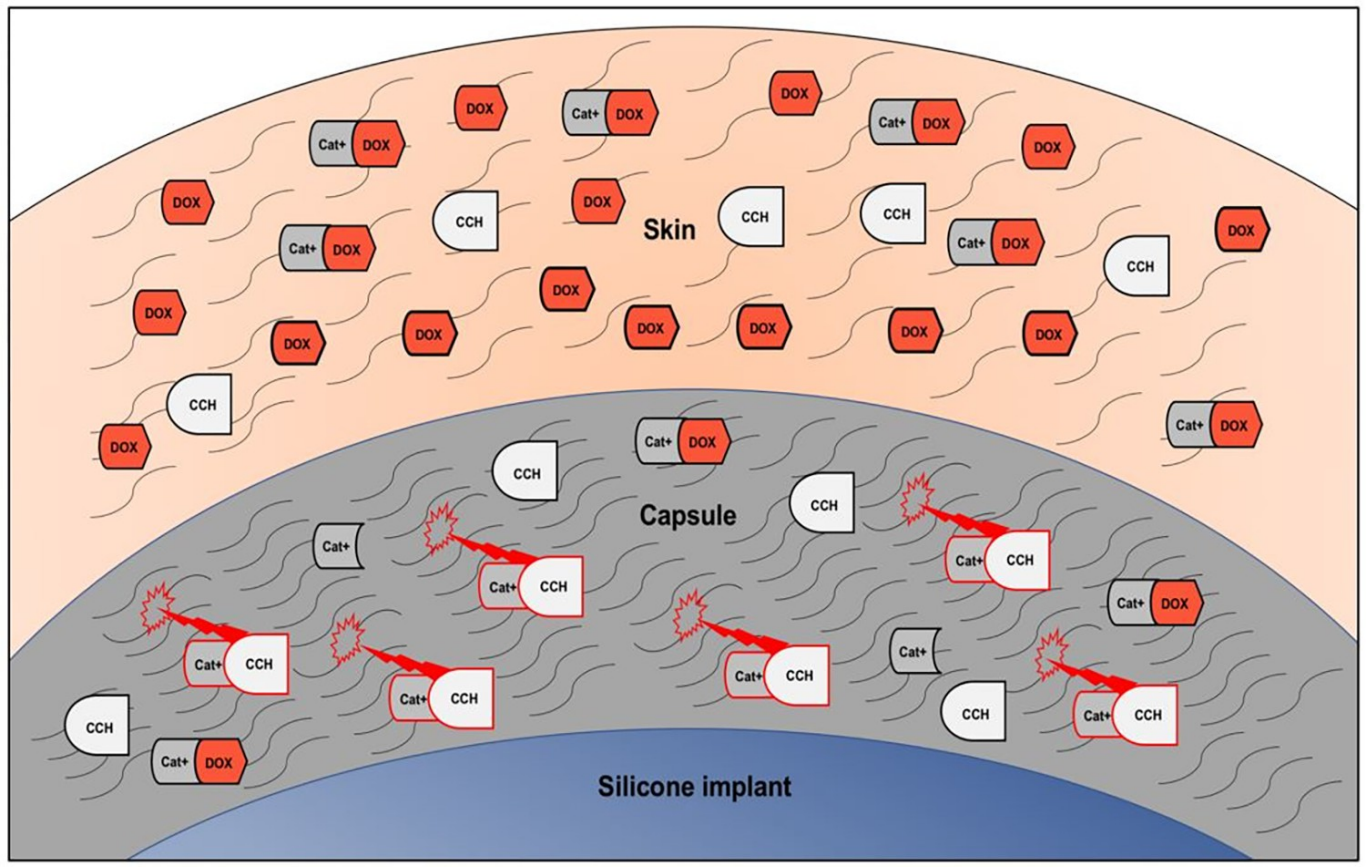
Postulated mechanism of action. Schematic description of the potential mechanism of action for skin protection after CCH-injection through doxycycline. CCH (collagenase of the bacterium C. histolyticum) needs to bind metal cations (Cat+) for active degradation of collagen, which is the main component of the fibrotic capsule around silicone implants. Doxycycline (DOX) accumulates in the skin and acts as a metal cation chelator. CCH-molecules diffusing into the skin from the capsule are inactivated, due to the competitive binding of Cat+ to DOX-molecules. This leads to reduction of CCH-induced collagen degradation in the skin and thus protection from CCH-related skin affection.

We hypothesize, that oral doxycycline administration prior to CCH-injections can prevent collagenase-induced skin affection. The postulated mechanism of action is displayed in Fig 1.

## Methods

### Study design and animal model

This study consists of in-vitro experiments to validate the inhibitory effect of doxycycline on CCH and in-vivo studies to evaluate the skin-protecting capability of doxycycline during CCH-treatment. This study was carried out in strict accordance with the guidelines for animal research. After protocol approval by the local animal use committee Rhineland-Palatine (Koblenz, Germany, G 17-7-017), 78 female Lewis rats weighing 175-200g (10–12 weeks) received one textured miniature silicone implant each (Polytech, Germany) to induce capsular fibrosis as previously described [29, 30]. Animals were purchased by Charles River Laboratories (Sulzfeld, Germany). Briefly, implants were inserted in a subcutaneous pocket on the back of each rat through a small paravertebral incision. Subsequently, a fibrotic capsule develops around the implant. 120 days later, this capsule was evaluated and processed for histological or biochemical analysis. During the first postoperative week, animals were monitored on a daily basis for signs of wound infection, pain and illness (fur, automutilation, scratching, behavioral changes, eyes). In case of obscurity a veterinarian was consulted. Additionally, animals were weighed. Subsequently, frequency of monitoring was reduced to two times a week. All animals were housed in groups of 3 in polycarbonate cages (Makrolon Typ IV) under pathogenfree conditions, with food and water provided ad libitum and automated day-night cycle of 12 hours. Room temperature and humidity were 20 ± 2°C and 55% ± 5%. Postoperative pain management was performed by administration of rimadyl at a dose of 5 mg/kg body weight every 12 hours.

Follow-up time of 120 days was chosen based on our previous experiments and for clinical translation purposes. According to Sengupta et al. one month of an adult rat´s life equates to 3 human years, and thus 120 days reflect a follow-up-period of approximately 12 human years [31]. This follow-up-period is well in line with clinical studies reporting capsular fibrosis incidence [32].

For in-vitro studies, capsules of 6 rats were excised on day 120 after insertion and 1x1cm tissue specimen were incubated for 24h with 0.9 mg/ml CCH (Xiaflex, Endo Pharmaceuticals, Malvern, USA) and 4 different doses of doxycycline (0, 5, 10 and 20mg/ml for study groups named CCH9, Doxy5, Doxy10 and Doxy20). CCH was dissolved in 0.9% sodium-chloride with 0.03% calcium-chloride-dihydrate. 0.9mg/ml CCH was chosen based on previous studies, which showed that injections with 0.9mg/ml CCH are highly effective in dissolving capsule tissue while leading to skin affection in 50% of cases [18]. The above-mentioned dilution series of doxycycline was selected to monitor potential dose-dependent inhibitory effects of doxycycline with 20mg/ml being the highest commercially available solution which then was gradually diluted. For dilution 0.9% sodium-chloride was utilized. Incubation was performed in an incubator (Heracell™, ThermoScientific, USA) at 36°C, 5% CO_2_ and 21% O_2_ for 24h. After incubation, capsule tissue was prepared for histology and collagen quantification.

For in-vivo studies, animals were divided into 6 groups with 12 rats each. Animals of groups named Doxy, DCCH3 and DCCH9 received two doses of 3mg/kg bw doxycycline (Lethal Dose 50% (LD50) value >1700mg/kg bw [33]) with oral gavage feeding tubes on day 118 and 119 post implant insertion. 3mg/kg bw doxycycline was selected based on recommended doses for clinical use in humans (250mg doxycycline for humans 80kg). Administration times were chosen 1 and 2 days prior to CCH application to built-up serum and tissue levels and to reflect a potential clinical treatment regimen. On day 120, CCH was injected at a dose of 0.3mg/ml (groups CCH3 and DCCH3) or 0.9mg/ml (groups CCH9 and DCCH9) into implant pockets as previously described [16, 18]. Both CCH concentrations were selected based on previous studies, showing skin affection in 17% (0.3mg/ml) and 50% (0.9mg/ml) of cases while effectively dissolving the fibrotic capsule [16, 18]. 0.9% sodium-chloride with 0.03% calcium-chloride-dihydrate served as solvent solution and was injected in groups CG and Doxy as control. 10 days after injection, animals underwent CT-scans followed by euthanasia using Narcoren over-dose (Boeringer Ingelheim Vetmedica, Germany) and resection of the silicone implant with surrounding capsule tissue. Subsequently, capsule specimen were prepared for histology, collagen assay and gene analysis. All measurements and assay evaluations were performed by an independent investigator blinded for the group allocation (SF).

**Fig 2:**
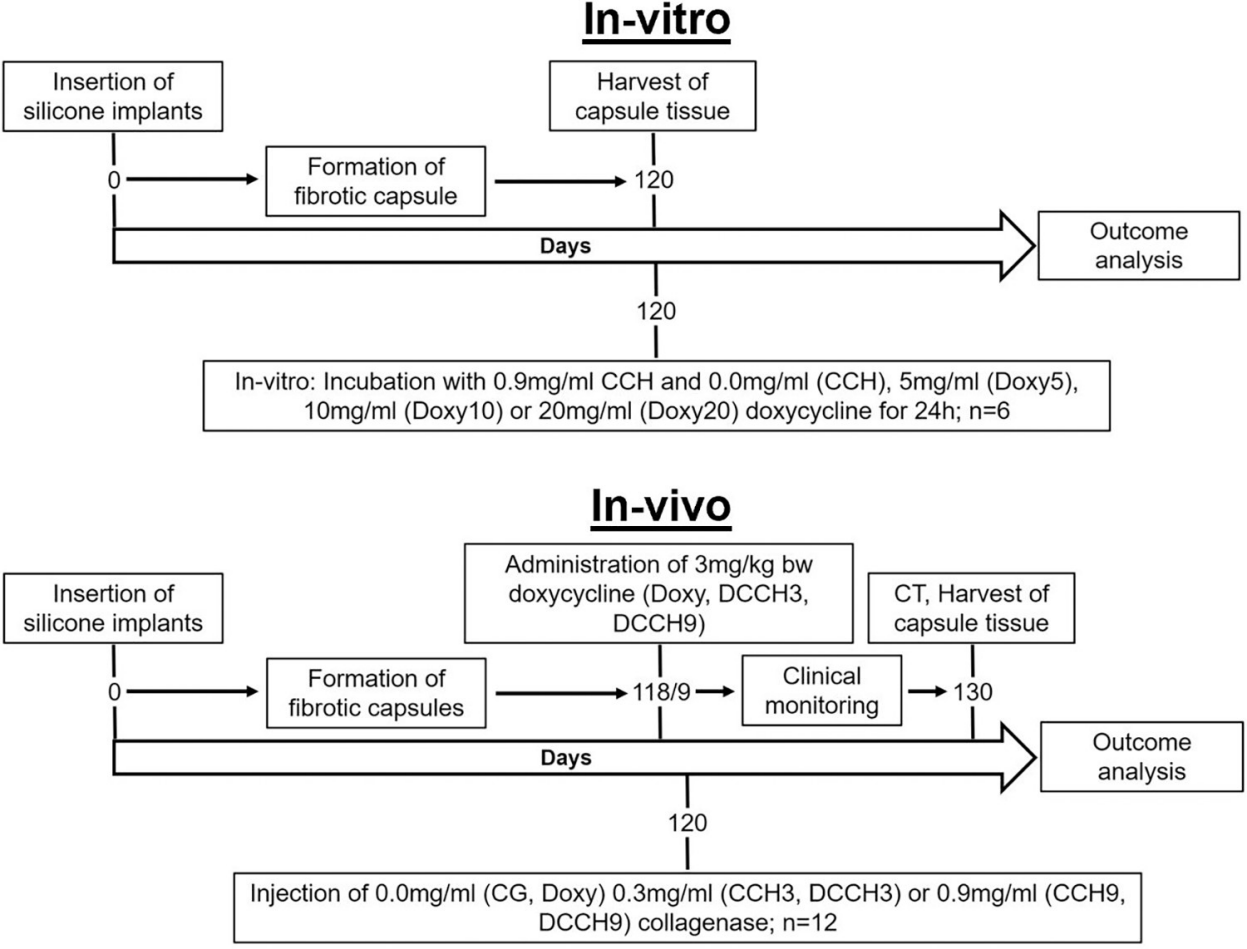
Illustration of study design. Study design for in-vitro and in-vivo experiments. CCH = collagenase of the bacterium C. histolyticum. Group size was n = 6 for in-vitro and n = 12 for in-vivo experiments. Outcome analysis included clinical monitoring, skin and capsule thickness and collagen density measurements, collagen content determination and gene expression analysis.

Table 1 and Fig 2 give an overview of the study design and all study groups including all group labels.

**Table 1:** Study groups of in-vitro and in-vivo experiments. In-vitro: Group names and corresponding concentrations of CCH and doxycycline for tissue incubation. In-vivo: Group names and corresponding oral doxycycline doses for administration on day 118 and 119 after implant insertion and corresponding CCH concentrations for injection into the implant pocket on day 120.

In-vitro								
**Group**	CCH9		Doxy5		Doxy10		Doxy20	
**CCH [mg/ml]**	0.9		0.9		0.9		0.9	
**Doxycycline [mg/ml]**	0.0		5.0		10.0		20.0	
**In-vivo**								
**Group**	CG	Doxy		CCH3	CCH9	DCCH3		DCCH9
**CCH [mg/ml]**	0.0	0.0		0.3	0.9	0.3		0.9
**Doxycycline [mg/kg bw]**	0	3		0	0	3		3

### Primary/Secondary outcomes

Primary outcomes of this study were evaluation of skin affection: signs for clinical changes in skin, histologic analysis of skin thickness and skin collagen density.

Secondary outcomes were thickness, collagen density and collagen content measurements of fibrotic capsules, as well as in-vivo CT-imaging and gene expression analysis.

### Histology

Following formalin fixation with phosphate-buffered 4% formaldehyde and paraffin-embedding, evaluation of capsule and skin thickness was performed by Masson trichrome staining. Briefly, thin sections were stained in Weigert´s iron hematoxylin solution (Merck, Germany) before incubation in Biebrich scarlet-acid-fuchsin solution (Merck, Germany). Slides then were differentiated in phosphomolybdic-phosphotungstic-acid solution and transferred to aniline-blue solution (Merck, Germany). After several washing steps, slides were mounted and cover-slipped. At 10 randomly chosen sites at 40x magnification, capsule thickness was assessed using the ImageJ software (V. 1.46, NIH, USA). Capsule boundaries were defined between the surface of the implant and the first tissue that is not part of the capsule, namely fat or muscle. For skin, thickness was measured between skin surface and subcutaneous fat, accordingly.

Additionally, density of collagen fibers was calculated by Picrosirius Red staining. Slides were deparaffinized and washed in an alcoholic dilution series. Subsequently, slides were stained in Picrosirius Red solution (abcam, UK) for 60 minutes, washed in acetic acid, mounted and cover-slipped. Through polarized light microscopy at 40x magnification, collagen fibers were registered at 4 randomly chosen sites. Quantification of collagen fiber density was performed by pixel counting with ImageJ and expressed as a percentage of the whole image pixel count.

All results of histologic evaluation are given as mean±standard deviation.

### Collagen quantification assay

Capsule collagen content was quantified by the Sircol Soluble Collagen Assay Kit (Biocolor, Northern Ireland) according to the manufacturer´s instructions. Tissue samples were weighed and incubated in 0.5M acetic acid with 0.1mg/ml pepsin overnight to isolate the collagen. Then, Sircol-Dye-Reagent was added for precipitation and staining of the collagen. After washing with Acid-Salt-Wash-Reagent, collagen pellets were re-suspended in Alkali-reagent and absorbance was measured at 555nm by spectrophotometer (SpectraMax Plus 384, Molecular Devices, USA). Collagen concentrations were calculated using a standard curve generated according to the manual. Results are given in μg collagen/g tissue and mean±standard deviation.

### Clinical evaluation

Animals were monitored for incidences of adverse effects like affection of skin, hematoma/seroma formation or reduction of general well-being daily after CCH injections until euthanasia. Skin affection was defined as any visible alterations of the rat’s skin in the area of the implant/CCH-injection (e.g. lacerations, perforations, abrasions). If visible or palpable signs of fluid accumulation around the implant occurred, animals were examined by high-resolution-ultrasound with a 48MHz transducer (Accutome, USA) to detect hematoma/seroma.

### Computed tomography (CT)

On day 130, six animals of each group were imaged with CT. Scans were conducted by a dual-source CT with a 2x192 slice detector (SOMATOM Force, Siemens Healthineers, Germany). Slice thickness was set to 0.4mm with a 0.2mm increment. Scans were analyzed with ImageJ (v. 1.46, NIH, USA). Thickness of fibrotic capsules was measured as distance from the implant surface to the skin. Results are given as mean±standard deviation (SD).

### Quantitative Real-Time PCR (qRT-PCR)

qRT-PCR analysis was performed as previously described [18]. Total RNA was extracted with the RNeasy-Mini-Kit (Qiagen, USA) and transcribed in cDNA by means of ABI-PRISM TaqMan reverse-transcription (Applied Biosystems, USA). Expression levels of pro-fibrotic and inflammatory genes (collagen I-IV, TGFβ1/3, CD-68, IL12, VEGFa) were determined with the ABI-PRiSM-7900HT-System (Applied Biosystems, USA) and normalized to the housekeeping gene β2-microglobulin. Outcomes are listed as relative quantitation (RQ).

### High-Performance-Liquid-Chromatography (HPLC)

For determination of doxycycline concentration, blood and skin samples were harvested on the day of CCH-injections of 4 animals of groups Doxy, DCCH3 and DCCH9. For extraction, a commercially available extraction cartridge (HLB Oasis Extraction cartridge, Waters Associates, UK) was used as described by Axisa et al. [27]. After final elution, samples were injected into the chromatograph (Waters 600 HPLC Pump, Waters GmbH, Germany). Analysis was performed at a wavelength of 350nm by means of Chemstation chromatographic software (Agilent Technologies, USA). Results are given as mean±standard deviation in μg/ml.

### Statistics

To reach allocation concealment, a numerical code independent of study groups was assigned to each tissue sample. All measurements were performed by investigators blinded for group allocation by only knowing the numerical code. Animals of each study group were housed in two distinct cages, allocated to the corresponding study group. Ranomization was achieved through randomn allocation of the study animals to different cages upon arrival from the vendor. Rats were marked by ear punches for later identification. Sample size calculation was based on our previous studies [14, 16–18]. Sample size calculation was performed by means of GPower software (v3.1.9.7) [34]. With the assumption of equal standard deviations, type I error <0.05, type II error <0.2 and an effect size of 1.1, the number of animals needed per group was 12.

Acquired data was statistically analyzed by means of Statistical Package for Social Science SPSS, v18.0 (SPSS Inc., Chicago, USA) using analysis of variance (ANOVA) for comparison of all groups with post-hoc testing with student´s t-test and Bonferroni-correction of significance levels to p <0.0125 (in-vitro) and 0,0083 (in-vivo).

## Results

All results are summarized in Table 2.

**Table 2:** Summary of results of all outcome parameters for all groups. Units are given in the first column. Results are given as mean ± standard deviation. For qRT-PCR, Col = Collagen.

**In-vitro**						
**Group**	CCH9	Doxy5	Doxy10		Doxy20	
Capsule thickness [μm]	109.0±12.8	125.6±38.6	139.4±16.4		156.8±16.6	
Capsule Density [%]	7.2±1.9	8.0±1.8	11.5±2.5		13.1±2.6	
Capsule collagen content [μg/g]	5392.8±512.3	5282.6±1061.5	6304.8±769.5		7216.5±1032.5	
**In-vivo**						
**Group**	CG	Doxy	CCH3	CCH9	DCCH3	DCCH9
Doxycycline serum/tissue levels [μg/ml]	-	2.2±0.8/1.2±0.8	-	-	2.6±0.72/1.4±0.9	2.4±0.7/1.4±0.4
Clinical skin affection [%], (n)	0% (0)	0% (0)	17% (2)	33% (4)	0% (0)	8% (1)
Skin thickness [μm]	680.4±62.0	675.7±107.5	648.6±189.2	560.1±61.6	649.4±85.3	578.7±86.1
Skin density [%]	29.8±6.7	30.7±8.1	25.1±6.1	19.2±3.8	27.8±2.8	25.3±3.8
CT capsule thickness [mm]	1.3±0.4	1,5±0.6	1.1±0.2	1.0±0.3	1.1±0.2	1.0±0.2
Capsule thickness [μm]	313±65.9	316.7±91.3	151.1±22.9	105.2±51.8	183.9±77.5	113.7±63.1
Capsule density [%]	23.6±5.9	25.5±6.6	12.1±5.2	7.9±3.6	13.9±4.5	12.1±3.7
Capsule collagen content [μg/g]	9479.5±734.1	9781.3±1109.8	7513.6±1394.5	5666±1121.1	6810.5±1072	6025.7±1005.5
qRT-PCR [relative quantitation compared to CG]	-	Col1:0.9±0.2	Col1:2±0.4	Col1:2.1±0.2	Col1:1.6±0.3	Col1:1.8±0.4
		Col2:0.9±0.3	Col2:1±0.3	Col2:1.6±0.2	Col2:1.3±2.4	Col2:1.1±0.5
		Col3:1±0.3	Col3:3.1±0.8	Col3:1.9±0.5	Col3:2.6±0.5	Col3:2.1±0.5
		Col4:1.1±0.3	Col4:1.4±0.4	Col4:2.3±0.3	Col4:2±0.6	Col4:1.9±0.5
		IL12:0.8±0.3	IL12:2.9±0.4	IL12:1.3±0.4	IL12:2.7±0.7	IL12:2.1±0.5
		CD68:2±0.3	CD68:3.1±0.7	CD68:3.2±0.4	CD68:3.2±0.6	CD68:3±0.7
		TGFβ1:0.8±0.5	TGFβ1:1.6±0.3	TGFβ1:2.5±0.6	TGFβ1:2.1±0.1	TGFβ1:1.9±0.2

### In-vitro

#### Doxycycline decreases CCH-induced reduction of capsule thickness dose-dependently

**Fig 3:**
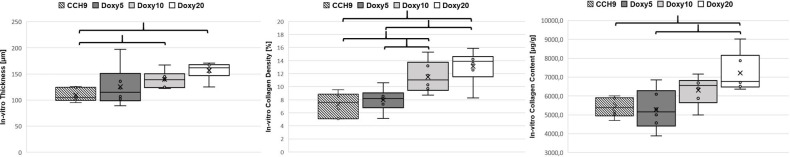
In-vitro results. Results of thickness, density and collagen content measurements. Mean values (X) are graphed on a box plot and individual data points are shown. *Density is measured as percentage of stained pixels in relation to the whole image pixel count*. Brackets indicate statistically significant differences (p < 0.0125). Group size: n = 6.

We observed a dose-dependent CCH-inhibition with mean values of 109.0±12.8μm (CCH9), 125.6±38.6μm (Doxy5), 139.4±16.4μm (Doxy10) and 156.8±16.6μm (Doxy20). Thickness of groups Doxy10 and Doxy20 was significantly bigger compared to those of CCH9 (p <0.0125). Results are shown in Fig 3.

#### Doxycycline decreases CCH-induced reduction of capsule density dose-dependently

In terms of collagen density, similar results were obtained. Doxycycline was able to reduce CCH activity in a dose dependent manner leading to a significantly lower density reduction in groups Doxy10 (11.5±2.5%) and Doxy20 (13.1±2.6%) compared to CCH9 (7.2±1.9%; p <0.0125). Additionally, capsules of Doxy20 had a significantly higher collagen density after incubation in comparison to Doxy5 (13.1±2.6% vs. 8.0±1.8%, p <0.0125). Results are shown in Fig 3.

#### Doxycycline decreases CCH-induced reduction of capsule collagen content dose-dependently

The addition of doxycycline yielded a significantly reduced CCH-induced collagen dissolution (CCH9: 5392.8±512.3μg/g vs. Doxy20: 7216.5±1032.5μg/g; p <0.0125). The dose-dependent inhibitory effect of doxycycline was apparent when comparing Doxy5 (5282.6±1061.5μg/g) with Doxy20 (p <0.0125). Mean collagen content in group Doxy10 was 6304.8±769.5μg/g with no significant differenced compared to the other study groups. Results are depicted in Fig 3.

### In-vivo–primary outcomes

#### Oral doxycycline administration reaches sufficient serum and tissue levels in all test groups

Serum levels of doxycycline were 2.2±0.8μg/ml (Doxy), 2.6±0.72μg/ml (DCCH3) and 2.4±0.7μg/ml (DCCH9) and did not differ significantly. Skin doxycycline concentration ranged from 1.2±0.8μg/ml (Doxy) and 1.4±0.4μg/ml (DCCH9) to 1.4±0.9μg/ml (DCCH3) with no significant differences.

#### Doxycycline leads to less clinical skin affection after CCH injection

**Fig 4:**
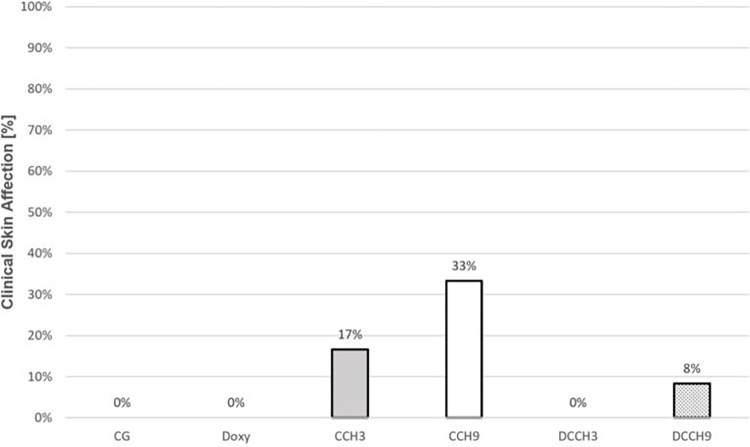
Results for clinical skin evaluation. Skin affection rates shown in % of the whole study groups (n = 12).

**Fig 5:**
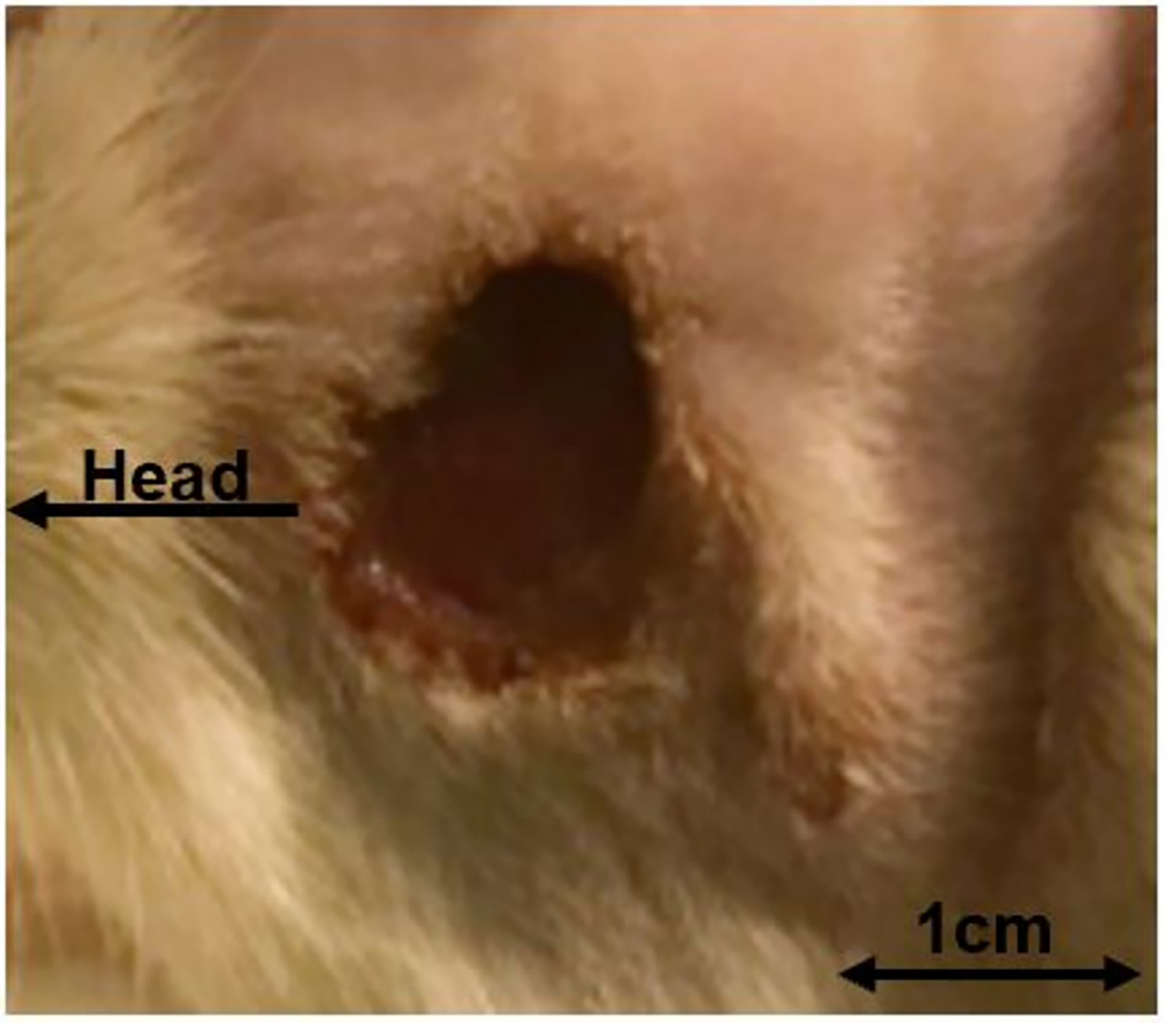
Skin affection. Skin affection of one animal in group CCH9 after 48 hours after injection of 0.9 mg/ml CCH.

Control groups CG and Doxy showed no signs of clinical skin affection (n = 0; 0%). After injection of CCH, clinical skin affection occurred in n = 2 (17%; CCH3) and n = 4 (33%; CCH9). Rats with doxycycline administration combined with CCH-injections displayed signs of clinical skin affection in n = 0 (0%; DCCH3) and n = 1 (8%; DCCH9). Results and an example of a clinical skin affection of group CCH9 are shown in Figs 4 and 5.

#### High-dose CCH induces skin thinning

**Fig 6:**
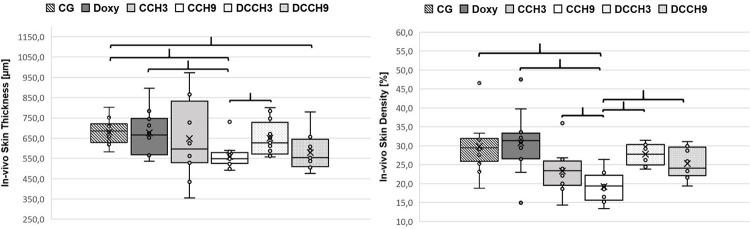
In-vivo results of skin measurements. left) skin thickness and right) skin collagen density measurements. Mean values (X) are graphed on a box plot and individual data points are shown. Density is measured as percentage of stained pixels in relation to the whole image pixel count. Brackets indicate statistically significant differences (p < 0.0083). Group size: n = 12.

**Fig 7:**
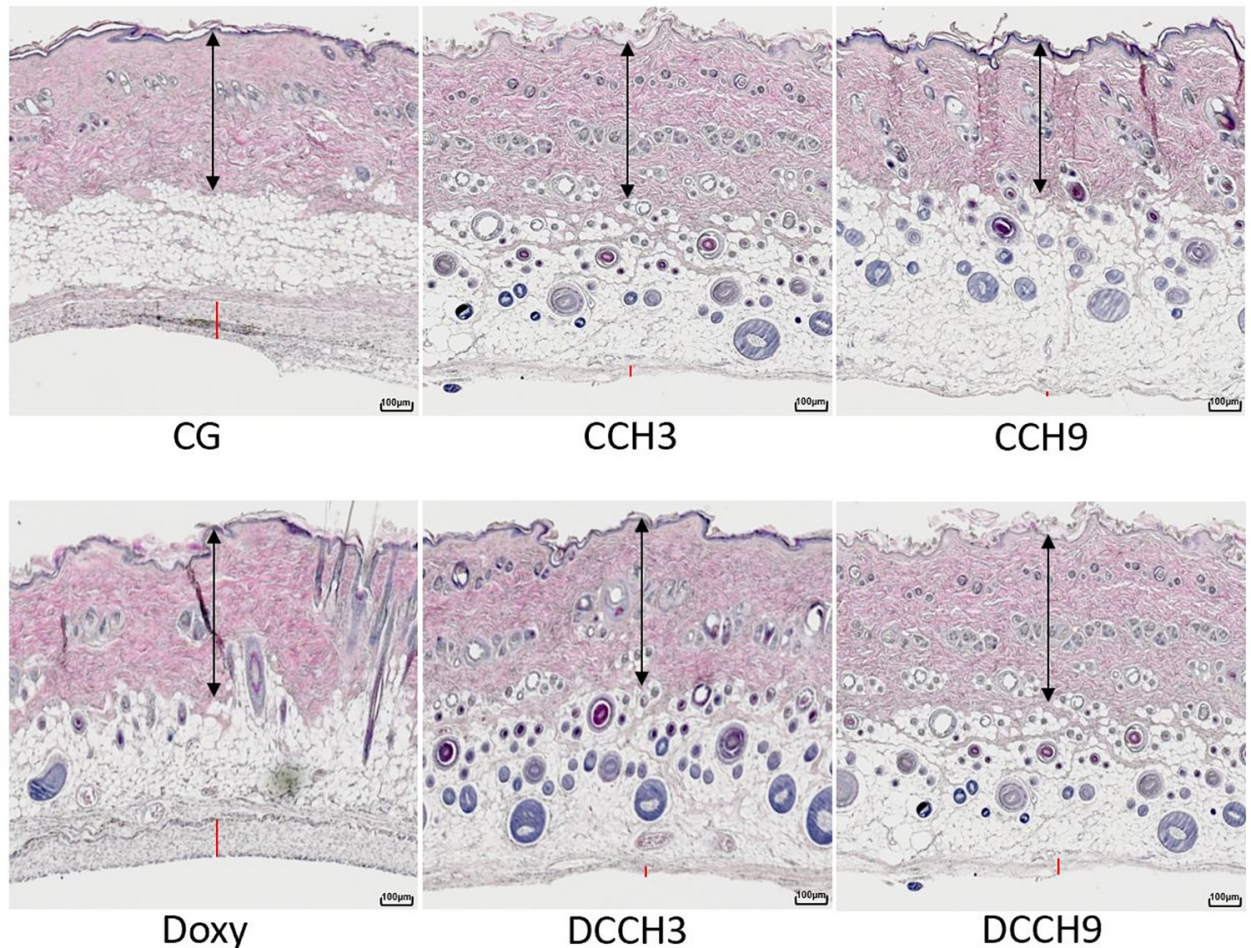
Example pictures of H&E staining of in-vivo study groups. Slides at 40x magnification with arrows indicating skin thickness and red lines indicating capsule thickness. Group size: n = 12.

For skin thickness, measurements showed a significant reduction after injection of the higher dose of CCH (0.9mg/ml) compared to both controls (CCH9: 560.1±61.6μm vs. CG: 680.4±62.0μm and Doxy: 675.7±107.5μm; p <0.0083). Additionally, rats of group CCH9 had significantly thinner skin compared to those of DCCH3 (649.4±85.3μm; p <0.0083). Skin in group DCCH9 (578.7±86.1) were significantly thinner compared to CG (p <0.0083). No other significant differences were observed (Fig 6). Example pictures of histologic outcomes are shown in Fig 7.

#### Doxycycline significantly prevents CCH-induced skin collagen density reduction

While CCH-injections at 0.3mg/ml did not decrease skin collagen density (CCH3: 25.1±6.1%), 0.9mg/ml CCH led to a significant reduction when compared with CG and Doxy (CCH9: 19.2±3.8% vs. CG: 29.8±6.7% and Doxy: 30.7±8.1%; p <0.0083). CCH9 showed significantly less collagen density compared to DCCH3 (27.8±2.8%) and CCH3 (p <0.0083).

After doxycycline administration, 0.3mg/ml and 0.9mg/ml CCH (DCCH3: 27.8±2.8% and DCCH9: 25.3±3.8%) were not able to decrease skin collagen density compared to the control groups CG and Doxy. Doxycycline prevented skin dissolvement leading to significantly higher residual skin collagen density when 0.9mg/ml CCH was used (DCCH9 vs. CCH9; p <0.0083).

### In-vivo–secondary outcomes

#### CT-Evaluation did not detect statistically significant differences between groups

ANOVA did not reveal statistically significant differences for CT-evaluation (Table 2). Capsule thicknesses as measured by CT were 1.3±0.4mm (CG), 1,5±0.6mm (Doxy), 1.1±0.2mm (CCH3), 1.0±0.3mm (CCH9), 1.1±0.2mm (DCCH3) and 1.0±0.2 (DCCH9).

#### Doxycycline does not interfere with the therapeutic CCH-effect

**Fig 8:**
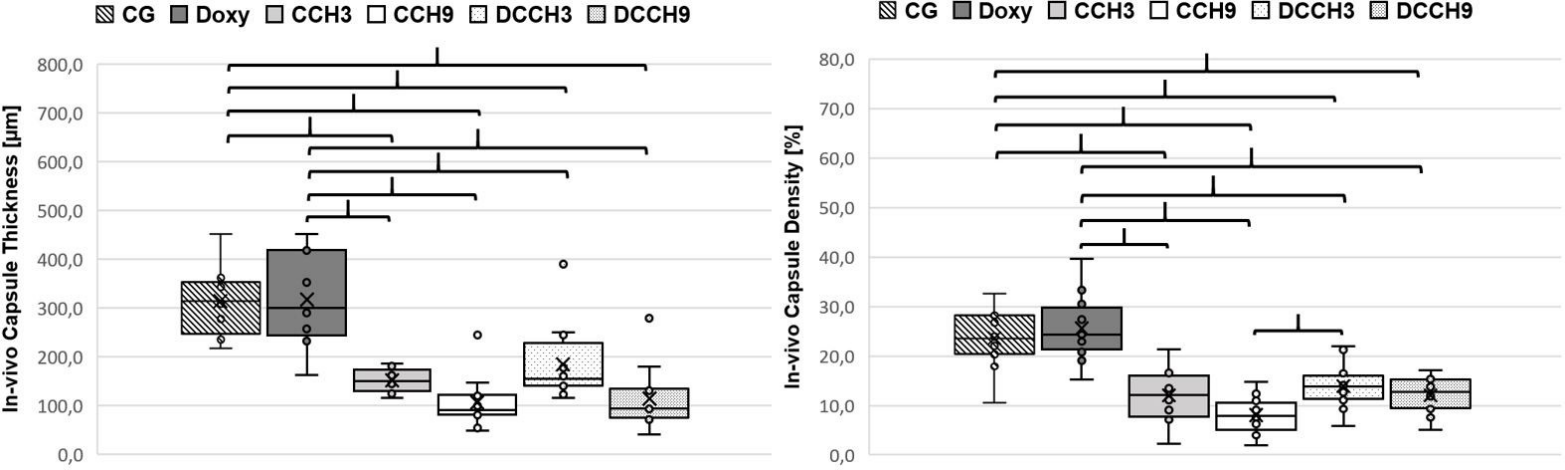
In-vivo results of capsule measurements. left) capsule thickness and right) capsule collagen density measurements. Mean values (X) are graphed on a box plot and individual data points are shown. Density is measured as percentage of stained pixels in relation to the whole image pixel count. Brackets indicate statistically significant differences (p < 0.0083). Group size: n = 12.

Compared to the control groups CG (313.0±65.9μm) and Doxy (316.7±91.3μm), capsule thickness was significantly lower after application of either 0.3mg/ml and 0.9mg/ml with or without prior doxycycline administration (CCH3: 151.1±22.9μm, CCH9: 105.2±51.8μm, DCCH3: 183.9±77.5μm, DCCH9: 113.7±63.1μm; p <0.0083). Results are shown in Fig 8.

Furthermore, CCH injections lead to significantly reduced collagen density in all test groups (CCH3: 12.1±5.2%, CCH9: 7.9±3.6%, DCCH3: 13.9±4.5%, DCCH9: 12.1±3.7%) when compared to both controls (CG: 23.6±5.9% and Doxy: 25.5±6.6%; p <0.0083; Fig 8). 0.9mg/ml CCH led to a significantly greater reduction of collagen within capsules (CCH9 vs DCCH3; p <0.0083). Administration of doxycycline alone did not lead to alterations in collagen density (CG vs. Doxy; p >0.0083).

**Fig 9:**
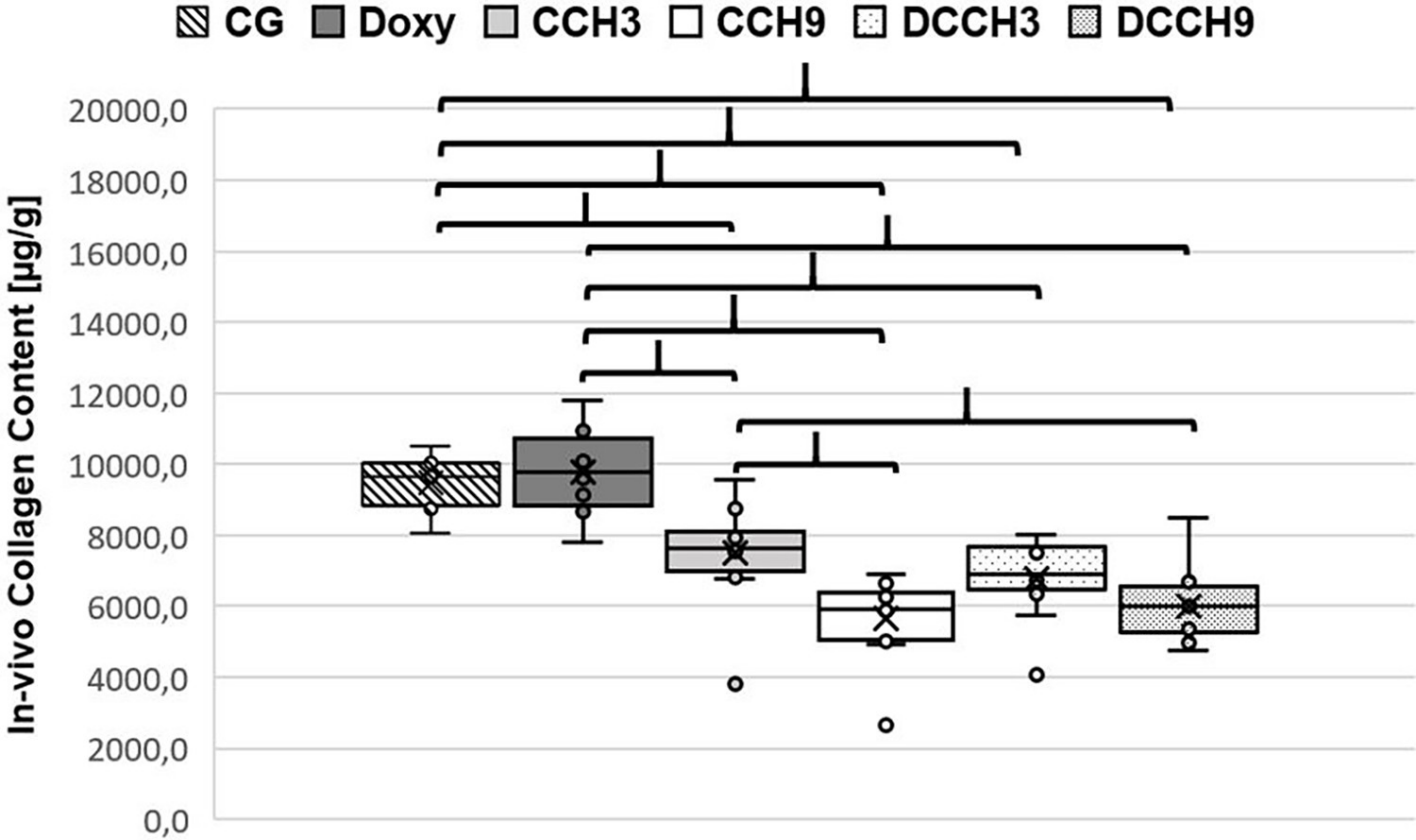
Results of in-vivo collagen quantification. Mean values (X) are graphed on a box plot and individual data points are shown. Brackets indicate statistically significant differences (p < 0.0083). Group size: n = 12.

Collagen content of capsules in all groups receiving CCH injections was significantly lower compared to both control groups (CCH3: 7513.6±1394.5μg/g, CCH9: 5666±1121.1μg/g, DCCH3: 6810.5±1072μg/g, DCCH9: 6025.7±1005.5μg/g vs. CG: 9479.5±734.1μg/g and Doxy 9781.3±1109.8μg/g; p <0.0083; Fig 9). Additionally, injection of 0.9mg /ml CCH led to a significantly stronger dissolvement of capsule tissue when compared to 0.3mg/ml (CCH9 and DCCH9 vs CCH3; p <0.0083).

#### CCH-treatment induces overexpression of pro-fibrotic genes

**Fig 10:**
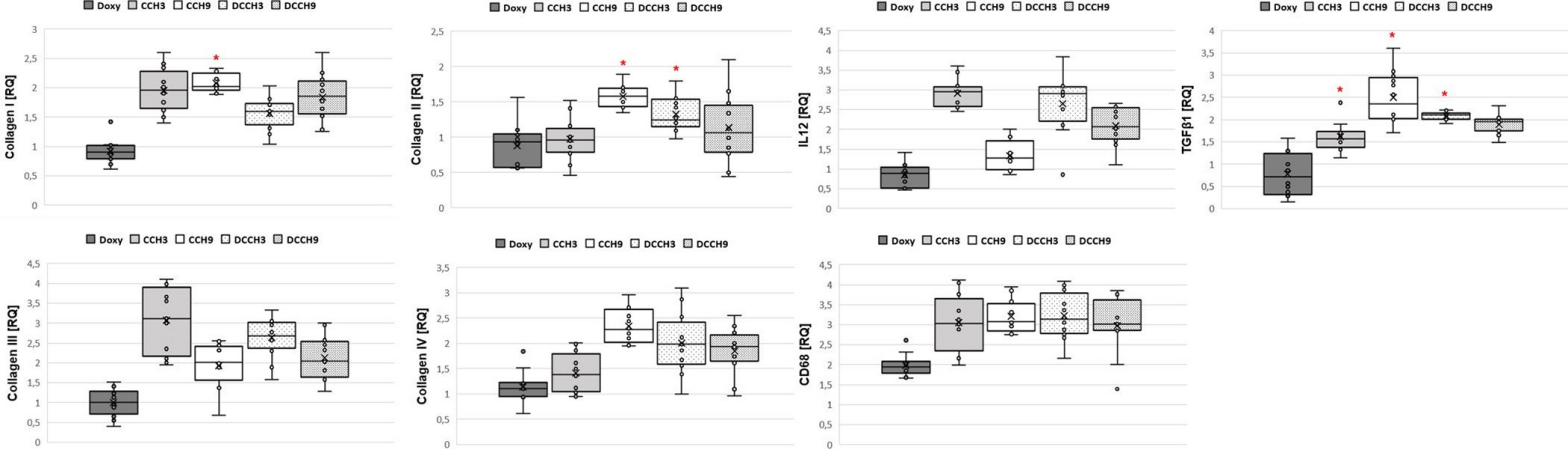
Results of gene expression analysis. Mean values (X) are graphed on a box plot and individual data points are shown. Red Asterix denotes statistically significant differences of study groups compared to control group CG. Group size: n = 12.

qRT-PCR analysis revealed a significant overexpression of collagen I after injection of 0.9mg/ml CCH (Fig 10). This was accompanied by a significant increase in collagen II expression in groups DCCH3 and CCH9. Furthermore, injection of CCH led to a significant overexpression of TGFβ1 in groups CCH3 (1.6±0.3), DCCH3 (2.1±0.1) and CCH9 (2.5±0.6) compared to the untreated control (p <0.0083). For the remaining genes, no significant differences were observed.

## Discussion

This study demonstrates that doxycycline is effective in reducing CCH-induced skin affection in an experimental model. This was determined by clinical evaluation, histology and chemical analysis.

This study presents a first benchmark that doxycycline might be capable of protecting the patient´s skin from CCH-related adverse effects. This gains even more interest as doxycycline is a readily available, FDA-approved drug. A clinical model would be a preventative oral or topical application of doxycycline in patients with M. Dupuytren, M. Ledderhose, M. Peyronie or capsular contracture two days prior to CCH-injections to induce a protective barrier within the skin. As described above, this might lead to a local inhibition of CCH in the skin without reducing the therapeutic effect of CCH. In terms of treatment costs of this proposed approach, doxycycline pre-treatment would cost around 20–50$ with a single CCH-injection amounting to 3–5000$ without personnel costs. In our opinion, avoiding further physical harm and additional surgical procedures through this minimal-invasive CCH-treatment combined with doxycycline skin protection would be the most important advantage and benefit for the patient.

The key concept of the treatment of M. Dupuytren and M. Peyronie as well as the potential treatment of capsular fibrosis around silicone implants with CCH is the localized effect on pathologic tissue. While CCH is not capable of distinguishing between healthy and pathologic collagen, Badalamente and Hurst were able to demonstrate that the CCH does not diffuse into adjacent tissue after injection [35]. This is in accordance with histological evaluation performed by Gelbard et al.. The authors found that the effect of CCH-injections was limited to the site of injection [36]. In our experiment, we did not find any signs for accidental paravasation into adjacent tissue. If present, skin affection was located in the area of the initial CCH-application. In this context, the risk for systemic reactions needs to be discussed. Both CCH and doxycycline have an overall good side-effect profile [37–40]. While the potential risk for direct injections of CCH into blood vessels exists, Sussman et al. showed that even CCH-concentrations considerably higher than those used in the clinical setting do not lead to system reactions [41, 42].

While the presences of wounds and postoperative swelling is widely accepted as a consequence of surgery and not considered a complication, skin damage induced by CCH-injections is the most common complication of this minimal-invasive procedure [23, 40]. Skin lesions after CCH-therapy occur in up to 16.2% of all cases; this is 5.7 times higher when compared to skin-associated adverse effects after surgery [23]. The majority of wound lesions after enzymatic treatment can be treated by normal wound therapy, healing within a time frame of 10–21 days, yet, severe wound healing impairments can persists in some cases [11].

There are several limitations to our study. The presented results were obtained in an in-vitro and in-vivo rodent model, with different anatomy and reactions to pharmaceuticals compared to humans. Due to the lack of an experimental model of M. Dupuytren or M. Peyronie, the presented analysis is based on an animal model on capsular contracture. It is important to note that although we tried to mimic clinical conditions, rodent skin is only a third as thick and 10-times more permeable as compared to human skin and therefore, results cannot directly be translated into a clinical setting [43, 44].

However, as collagen is the most homogenous protein among mammals, a similar effect of the combination of doxycycline and CCH on human tissue can be expected [17, 45].

Furthermore, our follow-up time after doxycycline and CCH administration was only 10 days. Although a different study by our group showed persistent positive outcomes on capsule tissue in the long-term, long-term effects regarding safety and recurrence of fibrosis are still unknown and need to be investigated [16]. For M. Dupuytren, recurrence rates are up to 47% within 5 years, which seems high but is comparable to recurrence rates after surgical treatment [40]. Regarding CCH-induced skin affection, it most commonly occurs during the first 48 hours after injection, as the collagenase is expected to become inactive in vivo [12]. In our earlier studies investigating the effect of CCH on capsule tissue, we utilized MRI and Ultrasound for in-vivo capsule analyzation. However, we changed this method of in-vivo examination in our more recent studies due to two reasons [14–17]. First, we found that while ultrasound is a useful tool in humans, ultrasound-evaluation of capsule formation around silicone implants in rats is very imprecise and not suitable for detection of thickness differences between animals. We rather changed our protocol and utilized ultrasound only for detection of fluid accumulation (e.g. seroma) within the capsule. Second, MRI based evaluation of capsule tissue is a very long and stressful procedure for the animals. In an unpublished study of our group we compared different imaging modalities (ultrasound, MRI, CT) in this animal model and compared it with histological finding. We did not find significant differences between the correlation of MRI or CT scan with histology. Therefore, we started to utilize CT scans for in-vivo capsule evaluation as this is a fast and significantly less harmful procedure for the animals.

Before implementing the application of doxycycline to prevent skin lacerations during CCH-injections into clinical practice, a pilot study in human patients will be necessary.

## Conclusion

The use of doxycycline as a skin protecting agent for collagenase treatment proved to be effective in the presented animal model. Its unique capabilities of accumulation in skin, inhibition of bacterial collagenase, tolerability and already existing FDA approval make doxycycline a promising adjunct to prevent collagenase-induced skin affection–not only in Dupuytren’s and Peyronie’s disease but also in the potential treatment of capsular contracture after silicone breast implants. However, these results were obtained in a rodent model and further validation for humans is required.

## Supporting information

S1 Dataset Minimal dataset. (XLSX)
